# Respiratory and Intramuscular Immunization With ChAdOx2-NPM1-NA Induces Distinct Immune Responses in H1N1pdm09 Pre-Exposed Pigs

**DOI:** 10.3389/fimmu.2021.763912

**Published:** 2021-11-03

**Authors:** Eleni Vatzia, Elizabeth R. Allen, Tanuja Manjegowda, Susan Morris, Adam McNee, Veronica Martini, Reshma Kaliath, Marta Ulaszewska, Amy Boyd, Basudev Paudyal, Veronica B. Carr, Tiphany Chrun, Emmanuel Maze, Ronan MacLoughlin, Pauline M. van Diemen, Helen E. Everett, Teresa Lambe, Sarah C. Gilbert, Elma Tchilian

**Affiliations:** ^1^ Enhanced Host Responses, The Pirbright Institute, Pirbright, United Kingdom; ^2^ Nuffield Department of Medicine, The Jenner Institute, University of Oxford, Oxford, United Kingdom; ^3^ Aerogen Ltd., Galway, Ireland; ^4^ Animal and Plant Health Agency (APHA)-Weybridge, Addlestone, United Kingdom

**Keywords:** influenza A, pig, vaccine, pre-exposure, pdmH1N1, aerosol, intranasal, intramuscular

## Abstract

There is a critical need to develop superior influenza vaccines that provide broader protection. Influenza vaccines are traditionally tested in naive animals, although humans are exposed to influenza in the first years of their lives, but the impact of prior influenza exposure on vaccine immune responses has not been well studied. Pigs are an important natural host for influenza, are a source of pandemic viruses, and are an excellent model for human influenza. Here, we investigated the immunogenicity of the ChAdOx2 viral vectored vaccine, expressing influenza nucleoprotein, matrix protein 1, and neuraminidase in H1N1pdm09 pre-exposed pigs. We evaluated the importance of the route of administration by comparing intranasal, aerosol, and intramuscular immunizations. Aerosol delivery boosted the local lung T-cell and antibody responses, while intramuscular immunization boosted peripheral blood immunity. These results will inform how best to deliver vaccines in order to harness optimal protective immunity.

## Introduction

Influenza virus infection remains a global health threat to humans, and animal influenza A virus is an important zoonotic pathogen with pandemic potential. There is an urgent need to develop vaccines that provide broader protection and decrease the need of annual vaccination. Resolution of two major issues should make rational immunization design easier. The first is that most humans or animals have already encountered influenza virus, and this may bias subsequent immune responses toward the virus epitopes from the first exposure (antigenic sin) which may decrease vaccine-induced protection (1, 2). Therefore, the immunological impact of prior influenza virus exposure on vaccine efficacy needs to be taken into account. The second is that although local immune responses are critical for protection against mucosal infection, whether local immunization offers a real advantage remains to be proven.

Most people are infected with influenza viruses once every 5 years, and this pre-existing immunity can significantly impact vaccine efficacy (3, 4). Cross-reactive immunity acquired by prior seasonal influenza infections is due to T-cell responses to conserved internal antigens and antibodies to conserved epitopes of the hemagglutinin (HA) and neuraminidase (NA) (5). It is well established that T-cell responses to conserved influenza A proteins such as the nucleoprotein (NP) and matrix protein 1 (M1) acquired by infection with influenza virus offer protection against symptomatic disease upon re-infection (6–9). We previously demonstrated that these T-cell immune responses can be boosted by intramuscular immunization with replication-deficient viral vectors Chimpanzee Adenovirus Oxford (ChAdOx) and Modified Vaccinia virus Ankara (MVA) expressing NP and M1 in humans (10). We further showed that inclusion of a third antigen, the HA, in ChAdOx-NPM1-HA and MVA-NPM1-HA significantly reduced virus shedding in pigs after prime boost vaccination against homologous H1N1pdm09 virus challenge (11). Recent research has underlined the role of anti-NA antibodies, which are induced after natural influenza virus infection (12, 13). Current vaccine development focuses on the HA where the majority of neutralizing epitopes are found. However, neutralizing antibodies (nAbs) directed to the HA are often on regions that have high sequence diversity and, thus, may not generate cross protection. Therefore, the inclusion of NA as a component of the influenza vaccine may help provide robust and broad protection.

The route of immunization and induction of local immune response are critical for vaccine efficacy (14–16). Although it is clear that local respiratory immune responses and tissue-resident memory T cells (TRM) are best induced by local respiratory immunization or infection, it is not clear which part of the respiratory tract (RT) should be targeted for optimal protection. Two airway immunization strategies have been developed: local nasal spray and aerosol delivery targeting the lung. In humans, an aerosol measles vaccine has been successfully deployed in Mexico and a live attenuated influenza virus (LAIV) is given to children and adults as a nasal spray (17, 18). Aerosolized vaccines are also currently investigated for COVID-19 (19).

However, targeting the lower or upper respiratory tract (LRT or URT) has important safety and immunological implications (20, 21), and studies with measles (22), *Mycoplasma pulmonis* (23), tuberculosis (24), and influenza (25–28) indicate that nasal delivery and lung targeting elicit distinct immune responses. In contrast, parenteral intramuscular delivery induces a systemic response, although there are reports showing that antigen-specific T cell may traffic to the mucosal surfaces after parenteral immunization (29, 30).

Based on these considerations, it is critical to study how vaccines can be optimally delivered to the different areas of the respiratory tract (RT) in large animal models and humans and to consider the effect of prior virus exposure on immune responses to the vaccine. Pigs, like humans, are a natural host for influenza A virus and display similar clinical manifestations and pathogenesis, making them an excellent large animal model for studying influenza infection and new vaccine candidates (31). The lobar and bronchial anatomy of the pig lung is similar to that of humans: they share the same histological structure, epithelial lining, distribution of sialic acid receptors, and electrolyte transport (32). We have developed methods to target different parts of the RT and used scintigraphy *in vivo* to analyze the distribution of antigen in pigs (33). Furthermore, for the first time, we have identified porcine TRM and characterized their specificity, function, and distribution in the respiratory tract (34–36).

Here, we used these tools and the pig influenza virus model to determine how to target antigen delivery optimally to the respiratory tract to induce URT and LRT immunity. We evaluated the immunogenicity of ChAdOx2 expressing NP, M1, and NA after different routes of immunization: targeting the whole RT by aerosol, the URT intranasally, or systemic immunity by intramuscular immunization in H1N1pdm09 pre-exposed pigs.

## Material and Methods

### ChAdOx2 Viral Vectored Vaccines

ChAdOx2 is a replication-deficient (E1 and E3 deleted) simian adenovirus (37), which we engineered to express swine influenza A virus nucleoprotein (NP) and matrix 1 (M1) as a fusion protein (NPM1) and/or NA. The NP and M1 protein ORFs from A/swine/England/1353/2009 (GenBank accession numbers KR701098 and KR701100) fused together by a glycine linker were synthesized by GeneArt Gene Synthesis (Thermo Fisher Scientific). The neuraminidase (N2) from H3N2 strain A/sw/Ohio/A01354299/2017 (GenBank accession number MF801571) was synthesized by GeneArt Gene Synthesis. The influenza virus genes were inserted into a Gateway^®^ recombination shuttle plasmid (pENTR LPTOS), containing a human cytomegalovirus major immediate early promoter (IE CMV), which includes intron A and two tetracycline operator 2 sites, and the bovine growth hormone polyadenylation signal, either by homologous recombination using NEBuilder^®^ HiFi DNA assembly kit (New England Biolabs) or classical restriction enzyme cloning. A shuttle plasmid containing N2 linked to NPM1 *via* the 2A ribosome skipping sequence from foot and mouth disease virus (FMDV) was generated by a 3-fragment ligation using NEBuilder^®^ HiFi DNA assembly kit. Briefly, pENTR LPTOS-NA was digested with *Kpn*I the recognition sequence of which is upstream of the NA ORF, and this was joined to the *Hin*dIII–*Not*I fragment NPM1 from the shuttle plasmid pENTR LPTOS-NPM1 described above that contains homology to the shuttle vector at the 5′ end and 2A sequence which was amplified from a previous construct (38) using primers with 5′ homology to 3′ NPM1 excluding the stop codon and 3′ homology to the 5′ NA including the start codon.

BACs containing the ChAdOx2-NPM1, ChAdOx2-NA, or ChAdOx2-NPM1-2A-N2 were prepared by Gateway^®^ recombination between the ChAdOx2 destination DNA BAC vector as previously described (39) and the shuttle plasmids containing the influenza virus gene expression cassettes using standard protocols resulting in the insertion of the expression cassette at the E1 locus. The ChAdOx2 adenovirus genomes were excised from the BAC using unique *Pac*I sites flanking the adenovirus genome sequence. ChAdOx2-NPM1, ChAdOx2-N2, or ChAdOx2-NPM1-2A-N2 viral vectors were rescued in T-REx™ cells (Invitrogen, Cat. R71007), a derivative of HEK293 cells which constitutively express the Tet repressor protein and prevent antigen expression during virus production. The resultant viruses, ChAdOx2-NPM1, ChAdOx2-N2, or ChAdOx2-NPM1-2A-N2, were purified by CsCl gradient ultracentrifugation as described previously (40). The titers were determined on T-REx™ cells using anti-hexon immunostaining assay based on the QuickTiter™ Adenovirus Titer Immunoassay kit (Cell Biolabs Inc.).

### Vaccine and Virus Challenge

Pigs were infected with the swine isolate H1N1 A/swine/England/1353/2009 (pH1N1), provided by the Animal and Plant Health Agency (APHA) (DEFRA swine influenza A virus surveillance program SV3041).

### Animals, Influenza Challenge, and Immunization

The animal experiment was approved by the ethical review process at APHA and followed the UK Government Animal (Scientific Procedures) Act 1986. Twenty, 7-week-old female Landrace × Large White pigs were pre-screened for the absence of influenza A virus antibody reactivity by HAI with four swine influenza A virus antigens: H1N1pdm09, H1N2, H3N2, and avian-like H1N1. One week after acclimatization, all 20 pigs were inoculated intranasally with 7.76 × 10^6^ TCID_50_ pH1N1 in a total of 4 ml (2 ml per nostril) using a mucosal atomization device (MAD, Wolfe-Tory Medical). Following H1N1pdm09 (pH1N1) challenge, daily nasal swabs were collected for 7 days to assess the virus load by plaque assays as previously described (34).

Four weeks post-pH1N1 challenge, the animals were randomly assigned to four groups and immunized with the same dose of 5 × 10^8^ infectious units (IU) ChAdOx2-NPM1-NA as follows: 1) intramuscularly (IM) with 1 ml administered in each trapezius muscle behind the ear; 2) aerosol (AE) with 1 ml administered over 2–5 min using an Aerogen Solo vibrating mesh nebulizer (Aerogen, Dangan, Galway, Ireland) (33); 3) intranasally (IN) with 300 µl per nostril administered with a MAD, with the aim of restricting the vaccine to the upper respiratory tract (33); and 4) unimmunized controls. The dose of 5 × 10^8^ IU of ChAdOx2-NPM1-NA vaccine was consistent with our previous studies with ChAdOx-vectored vaccines in pigs which had been found to be effective (11, 41). This is a much lower dose than that used in mice but is nevertheless effective in large animals and humans. The animals in the IN and AE groups were anesthetized with a mixture of 5 mg/kg Zoletil (2.5 mg/kg of tiletamine + 2.5 mg/kg of zolazepam) and 0.05 mg/kg Domitor (medetomidine).

Blood was collected weekly to assess immune responses of peripheral blood mononuclear cells (PBMC) and antibodies in the serum. Four weeks post-immunization, all animals were humanely culled with an overdose of pentobarbital sodium anesthetic. Blood, bronchoalveolar lavage (BAL), spleen, tracheobronchial lymph nodes (TBLN), prescapular LNs, retropharyngeal LNs, and nasal turbinates (NT) were collected and processed as described before (34, 35).

### Serological Assays

Endpoint titer ELISAs and microneutralization (MN) assays for pH1N1 and H3N2 viruses were performed as described before (36). For the ELISA, 96-well microtiter plates (Nunc MaxiSorp, Sigma Aldrich, UK) were coated overnight with either 1 μg/ml recombinant NA protein (N2) (sequence matched to the vaccine antigen, GenBank accession number: ATE49827, produced by The Native Antigen Company) or with pH1N1 or H3N2 viruses (1 × 10^6^ PFU/ml). Plates were blocked with 200 μl blocking solution of 4% (w/v) milk powder (Marvel) in PBS, supplemented with 0.05% Tween-20 (PBS-T) for 2 h at room temperature. For the ELISA, serum, BAL, and nasal swabs at starting dilutions of 1:20, 1:2, and 1:4, respectively, were serially two-fold diluted in PBS-T with 4% (w/v) milk powder and added to the wells for 1 h on a rocking platform. The plates were washed three times with PBS-T and 100 μl of horseradish peroxidase (HRP)-conjugated secondary antibody diluted in PBS-T with 4% milk powder was added, and the plates were incubated for 1 h at room temperature. IgG and IgA were detected using goat anti-pig IgG-HRP or goat anti-pig IgA-HRP polyclonal Abs (Bio-Rad Antibodies). The plates were washed four times with PBS-T and developed with 100 µl/well of 3,3′,5,5′-tetramethylbenzidine (TMB) High Sensitivity substrate solution (BioLegend, London UK). After 5 min, the reaction was stopped with 100 µl 1 M sulfuric acid and the plates were read at 450 and 570 nm with the Cytation3 Imaging Reader (Biotek, Swindon, UK).

For the MN assays, the starting dilution for serum was 1:10 and 1:2 for BAL and nasal swabs. Fifty microliters of serially diluted samples were incubated in 96-well flat-bottomed plates with an equal volume containing 1 × 10^6^ PFU/ml pH1N1 or H3N2. After 2 h of incubation at 37°C, MDCK SIAT-1 cells in VGM were added at 3 × 10^4^ cells/well to the serum/virus mixtures and incubated for a further 18 h at 37°C. Supernatants were removed and cells fixed with 4% paraformaldehyde for 30 min at 4°C. The cells were washed twice with PBS and 20 mM glycine before 50 µl/well of permeabilization buffer (PBS, 20 mM glycine, 0.5% Triton X-100) was added for 20 min at room temperature. The cell monolayer was washed twice with PBS and stained with 50 µl/well anti-IAV NP mAb (Clone: AA5H, Bio-Rad Antibodies, Abingdon, UK) for 1 h at room temperature. The cells were washed again with PBS followed by 50 µl/well goat anti-mouse IgG horseradish peroxidase (HRP)-conjugated secondary antibody (Dako). After staining, the cells were washed twice with PBS and TMB substrate was added and incubated for 5 min, the reaction was stopped with 1 M sulfuric acid, and absorbance was measured at 450 and 570 nm (reference wavelength) on the Cytation3 Imaging Reader (Biotek, Swindon, UK).

### IFNγ ELISpots

Cryopreserved isolates from PBMC, BAL, TBLN, prescapular LN, and spleen cells were used to assay the frequencies of IFNγ-secreting cells. MultiScreen-HA ELISPOT plates (Merck Millipore) were coated with 0.5 mg/ml anti-pig IFNγ (clone P2G10; BD Pharmingen) diluted in carbonate buffer at 4°C overnight. The following day, the plates were washed four times with PBS (no Tween) and blocked for at least 1 h at 37°C with culture medium [RPMI 1640 with stable glutamine supplemented with 10% fetal calf serum (FCS), 100 U/ml penicillin, and 100 mg/ml streptomycin]. After four washes with PBS, cells resuspended in culture medium were seeded in triplicates at 3 × 10^5^ cells per well. The cells were simulated either with pH1N1 or H3N2 [multiplicity of infection (MOI) of 1], 3 µg/ml ConA (positive control, Sigma-Aldrich), culture medium (negative control), or with one of the following pools of peptides at a concentration of 2 µg/ml: NP1, NP2, M1, NA1, or NA2 (**Supplementary Table 1**). Lyophilized peptides were dissolved in DMSO and diluted in RPMI so that the final concentration of DMSO in the peptide pools was 0.1% which is considered safe for cells. We did not include DMSO in the medium control wells. The plates were incubated for 40 h in a 37°C, 5% CO_2_ incubator. The plates were washed with PBS, 0.05% Tween 20 and incubated for 1.5 h at room temperature with 0.25 mg/ml biotinylated anti-pig IFNγ detection Ab (clone P2C11, BD Pharmingen) diluted in PBS supplemented with 0.01% Tween 20 and 0.1% BSA, followed by a 1-h incubation at room temperature with streptavidin-alkaline phosphatase (1:2,000 in PBS, 0.01% Tween 20, 0.1% BSA, Roche, Mannheim, Germany). Spots were visualized after the addition of 5-bromo-4-chloro-3-indolyl phosphate/nitro blue tetrazolium substrate (100 μl/well, Sigma-Aldrich) according to the instructions of the manufacturer. The reaction was stopped using tap water and the spots were counted using the AID ELISPOT reader (AID Autoimmun Diagnostika). Results were expressed as the number of IFNγ-producing cells per 10^6^ cells after subtraction of the number of IFNγ-producing cells in medium control wells. Results from NP1 and NP2 or NA1 and NA2 were pooled and shown as NP and NA, respectively.

### Intracellular Cytokine Staining

Cryopreserved cells from BAL, spleen, nasal turbinates, and TBLN were thawed and seeded at 1 × 10^6^ cells per well. The cells were stimulated overnight with pH1N1 or H3N2 (MOI = 1) or medium as a control at 37°C and 5% CO_2_. Those stimulated with the NP2 or M1 peptide pools (2 µg/ml) were only incubated for 1 h before the addition of Brefeldin A (GolgiPlug™, BD Biosciences) as per the instructions of the manufacturer. In some wells, a cocktail of phorbol 12-myristate 13-acetate (PMA)/ionomycin (BioLegend) was added as a positive control at the same time as the GolgiPlug. Duplicate wells, each containing 1 × 10^6^ cells, were seeded for each stimulation condition. After 4 h of incubation at 37°C, the cells were centrifuged for 4 min, 1,500 rpm, washed twice with PBS, and analyzed for cytokine production using the antibodies listed in **Table 1**. Briefly, cells were stained with the primary Abs for surface staining and with Near-Infrared Fixable LIVE/DEAD stain (Invitrogen), for the identification of live cells. Following a 20-min incubation at 4°C, cells were washed twice, fixed, and permeabilized with BD Cytofix/Cytoperm (BD Biosciences) as per the instructions of the manufacturer. Cells were incubated for 30 min at 4°C with the directly conjugated cytokine antibodies, washed twice, and stained with the secondary rat anti-mouse IgG2a antibody for 20 min at 4°C. Finally, the cells were washed twice, resuspended in PBS, and analyzed using a MACSquant Analyzer 10 (Miltenyi). The frequency of cytokine production shown is after the subtraction of the frequencies found in medium control wells (unstimulated).

**Table 1:** Antibodies used for the intracellular cytokine staining.

Antigen	Clone	Isotype	Fluorochrome	Source of primary Ab	Details of secondary Ab
CD4	74-12-4	IgG2b	PerCP-Cy5.5	BD Biosciences	
CD8b	PPT23	IgG1	FITC	Bio-Rad Laboratories	
TNF	MAb11	IgG1	BV421	BioLegend	
IFNγ	P2G10	IgG1	APC	BD Biosciences	
IL-2	A150D 3F1 2H2	IgG2a	PE-Cy7	Thermo Fisher	Rat anti-mouse, IgG2a, BioLegend

### Statistical Analysis

Statistical analyses were performed using GraphPad Prism 8.1.2 (GraphPad Software, San Diego, CA, United States). The data sets were first analyzed for normality and then subjected to a two-way ANOVA and Bonferroni’s multiple comparisons test. When data sets were not normally distributed, they were subjected to a Kruskal–Wallis test and Dunn’s multiple comparisons test (**Figures 3B, C, E**, **3B**, **4A, B**, **5G, H**). For the data sets where a non-parametric test was performed, the median and interquartile range are shown, instead of the mean and standard error mean (SEM). Significant differences were either depicted on the graph or listed in **Tables 2**, **3** (*p < 0.05, **p < 0.01, ***p < 0.001, ****p < 0.0001). Until 28 days post-infection (DPI), all animals were treated identically and significant differences between the groups were not identified.

**Table 2:** Significant differences between the four groups at the same time point after immunization.

Assays		Significant differences identified between groups post-immunization			
		Day 35	Day 42	Day 49	Day 56
ELISA IgG (**Figures 1B–D**)	H1N1	IM > AE (*p* = 0.001) IM > IN (*p* = 0.001) IM > C (*p* = 0.001)	IM > AE (*p* = 0.0002) IM > IN (*p* = 0.0001) IM > C (*p* = 0.0001) AE > IN (*p* = 0.006) AE > C (*p* = 0.01)	IM > IN (*p* = 0.0003) IM > C (*p* = 0.0002)	IM > IN (*p* = 0.02) IM > C (*p* = 0.02)
	H3N2	IM > AE (*p* = 0.0001) IM > IN (*p* = 0.0001) IM > C (*p* = 0.0001) AE > C (*p* = 0.03)	IM > AE (*p* = 0.0001) IM > C (*p* = 0.0001) AE > IN (*p* = 0.0033) AE > C (*p* = 0.0014)	IM > IN (*p* = 0.0002) IM > C (*p* = 0.0001) AE > IN (*p* = 0.0001) AE > C (*p* = 0.0001)	AE > IN (*p* = 0.05) AE > C (*p* = 0.05)
	N2	No significant difference (*p* > 0.05)	No significant difference (*p* > 0.05)	IM > AE (*p* = 0.0001) IM > IN (*p* = 0.0001) IM > C (*p* = 0.0001)	IM > AE (*p* = 0.0001) IM > IN (*p* = 0.0001) IM > C (*p* = 0.0001)
MN H3N2 (**Figure 1F**)		No significant difference (*p* > 0.05)	IM > AE (*p* = 0.0001) IM > IN (*p* = 0.0001) IM > C (*p* = 0.0001)	IM > AE (*p* = 0.001) IM > IN (*p* = 0.001) IM > C (*p* = 0.001)	No significant difference (*p* > 0.05)
ELISpots (**Figures 2A, D** **)**	NP	IM > AE (*p* = 0.04) IM > C (*p* = 0.02)	No significant difference (*p* > 0.05)	IM > AE (*p* = 0.003) IM > C (*p* = 0.002)	IM > AE (*p* = 0.03) IM > C (*p* = 0.009)
	pH1N1	No significant difference (*p* > 0.05)	No significant difference (*p* > 0.05)	IM > C (*p* = 0.01)	IM > C (*p* = 0.07)

**Table 3:** Significant differences in the same group between different time points after immunization for .

Peptides/groups	IM	AE	C
NP	35 > 28 DPI (*p* < 0.01) 49 > 28 DPI (*p* < 0.01)		
M1	35 > 28 DPI (*p* < 0.0001) 49 > 28 DPI (*p* < 0.0001) 56 > 28 DPI (*p* < 0.0001) 35 > 42 DPI (*p* < 0.05)		
NA	35 > 28 DPI (*p* < 0.05) 35 > 42 DPI (*p* < 0.01)	35 > 42 DPI (*p* < 0.05)	35 > 49 DPI (*p* < 0.05) 35 > 56 DPI (*p* < 0.001)

## Results

### Experimental Design, Virus Shedding, and Antibody Responses

We first considered the implications of generating a viral vectored vaccine expressing both the NA and the NPM1 fusion protein. We tested the immunogenicity of three ChAdOx2 vaccines in mice (ChAdOx2-NA, ChAdOx2-NPM1, and ChAdOx2-NPM1-NA). Mice were intramuscularly immunized with 8 × 10^7^ IU/ml of ChAdOx2 and euthanized 3 weeks later. T-cell responses to NPM1 were measured by IFNγ ELISpot, and antibody responses to NA were measured by NA-ELISA. We found that there were no significant differences in immune responses when comparing the bivalent vaccine construct ChAdOx2-NPM1-NA to the single antigen vaccines (**Supplementary Figure 1**). Because of the strong immune responses generated with the bivalent vaccine, we tested it in the context of influenza pre-exposure in a large natural host model.

**Figure 1:**
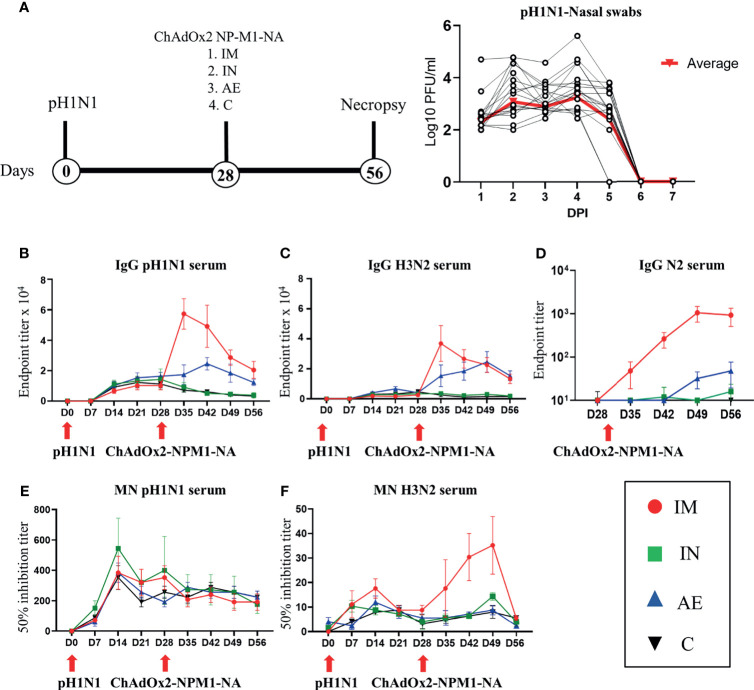
Experimental design, viral load, and systemic antibody responses. **(A)** Twenty pigs were infected with pH1N1 influenza A virus and, 4 weeks later, immunized with ChAdOx2-NPM1-NA intramuscularly (IM), intranasally (IN), or by aerosol (AE). Four weeks later, they were culled. Weekly blood samples were collected during the time course. Control (C) animals were infected but not immunized. Virus load was determined by plaque assay of daily nasal swabs (NS) obtained at the indicated days post-infection (DPI). Each black line represents one animal. The thick red line indicates the mean of 20 animals. **(B)** pH1N1-, **(C)** H3N2-, and **(D)** N2-specific IgG responses in serum were determined by ELISA at the indicated time points. **(E, F)** Serum-neutralizing titers over time were determined by microneutralization (MN) of **(E)** pH1N1 and **(F)** H3N2 viruses. The mean and standard error (SEM) is presented in each time point. The arrows below D0 and D28 indicate challenge of the pigs with pH1N1 and immunization with ChAdOx2-NPM1-NA, respectively. Significant statistical differences are listed in **Table 2**.

In order to assess the effect of influenza pre-exposure on vaccine immune responses, 20 pigs were infected intranasally with 7.8 × 10^6^ TCID_50_ of H1N1 A/swine/England/1353/2009 (pH1N1) (**Figure 1A**). Virus load after pH1N1 challenge was determined in daily nasal swabs (**Figure 1A**). In agreement with previous studies, virus load was detectable for 4 DPI followed by a sharp decline and was not detectable after 6 DPI (36).

Four weeks after the pH1N1 exposure, the pigs were divided into four groups of five animals and immunized with 5 × 10^8^ IU of ChAdOx2 virus vector expressing NPM1 and NA (ChAdOx2-NPM1-NA). The NP and M1 proteins were derived from H1N1 A/swine/England/1353/2009 with GenBank accession numbers KR701098 and KR701100, respectively, while the NA was from H3N2 A/swine/Ohio/A01354299/2017 with GenBank accession number MF801571. To evaluate the immunogenicity of parenteral and respiratory routes of immunization, ChAdOx2-NPM1-NA was administered IM by AE in order to reach the whole respiratory tract or IN, administered to the URT only. AE delivery by vibrating mesh nebulizer generated droplets of ~4.5 µm diameter capable of reaching the entire LRT as well as the URT (33). IN delivery was performed with a mucosal atomization device (MAD) generating droplets of ~80 to 100 µM diameter delivered in 300 µl volume in order to restrict the deposition of the vaccine to the URT. pH1N1-infected and unimmunized pigs were used as controls. The pigs were culled 4 weeks after immunization and tissues collected for the evaluation of immune responses in the respiratory tract, draining lymph nodes, spleen, and blood.

The antibody response after pH1N1 infection and ChAdOx2-NPM1-NA vaccination was evaluated in serum. Virus-specific IgG was measured by endpoint titer ELISA against H1N1pdm09 (pH1N1), which is an H1N1 influenza A virus strain from the 2009 pandemic, and H3N2 (**Figures 1B, C** **)**. Serum pH1N1-specific IgG was detectable from day 14, reaching a plateau at 21–28 DPI (1:12,800 and 1:13,056, respectively), and was similar in all animals until 28 DPI. Cross-reacting antibodies specific for H3N2 were also detectable and reached a peak of 1:3,600 at 21 DPI (**Figure 1C**). After immunization, Ab titers were highest to both pH1N1 and H3N2 in the IM group, reaching a peak of 1:58,000 and 1:38,000, respectively, at 35 DPI (7 days after immunization, *p* < 0.0001) (**Figures 1B, C** and **Table 2**). The titers declined over time but remained significantly higher compared with the control and IN groups until the end of the study for pH1N1 and until 49 DPI for H3N2. The AE immunization induced the second highest response to pH1N1 and H3N2 which peaked at 42 DPI (1:25,000) and 49 DPI (1:25,000), respectively, and was significantly higher compared with both the IN and control groups (*p* = 0.0057 and 0.0103, respectively, for pH1N1 and *p* < 0.0001 for both groups for H3N2). The H3N2 responses induced by AE immunization remained significantly higher compared to the IN and control groups until the end of the study (**Table 2**). Significant differences between groups are shown in **Table 2**. Intranasal immunization did not boost pH1N1 or H3N2-specific serum response (**Figures 1B, C** **)**.

We also measured the ELISA serum response to recombinant NA from H3N2 (N2), which peaked in the IM group at 49 DPI (**Figure 1D**). A lower N2 response but with a similar kinetic was detected in the AE group, while the response after IN immunization was minimal, with only a small increase at 56 DPI. The functional activity of the serum antibodies was evaluated by microneutralization (MN). MN serum titers peaked at 14 DPI for both pH1N1 and H3N2 and were maintained until 49 DPI (**Figures 1E, F** **)**. No increase in pH1N1 MN titer was observed after immunization by any route. Although H3N2 MN titers were lower compared with pH1N1, the IM immunization significantly boosted (**Table 2**) the response at 42 and 49 DPI (14 and 21 days post-immunization, mean of 50% inhibition 1:35).

**Figure 2:**
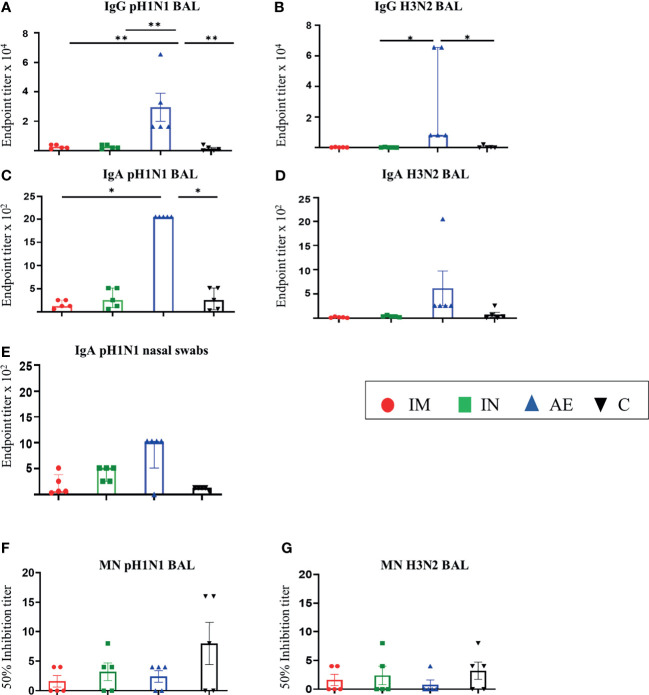
Mucosal antibody responses. **(A–D)** pH1N1- and H3N2-specific IgG and IgA responses in BAL and **(E) **pH1N1-specific IgA responses in nasal swabs were determined by ELISA 4 weeks after immunization. **(F, G)** BAL-neutralizing titers were determined by microneutralization (MN) of **(F)** pH1N1 and **(G)** H3N2 4 weeks after immunization. For graphs **(B, C, E)**, Kruskal–Wallis and Dunn’s multiple comparisons tests were performed, and the top of these bars indicates the median and the line the interquartile range. The rest of the graphs were analyzed by one-way ANOVA and Bonferroni’s multiple comparisons test, and the top of each bar indicates the mean and the line the standard error mean (SEM). Each symbol (circle, square, and triangles) represents one animal. Asterisks denote significance between indicated groups (*p < 0.05 and **p < 0.01).

In contrast, in the BAL fluid, AE immunization induced significantly higher IgG to pH1N1 compared to the other groups (**Figure 2A**), while AE induced H3N2 IgG and pH1N1 IgA, which were significantly higher compared to the IN and control group and the IM and the control group, respectively (**Figures 2B, C** **)**. Similarly, AE immunization induced a higher IgA H3N2-specific response and H1N1-specific pH1N1-specific response in BAL and nasal swabs on 56 DPI, but these were not significantly different to the other groups (**Figures 2D, E**). IgA responses to H3N2 in nasal swabs were also measured, but the responses were very low with no significant differences between groups (data not shown). The neutralizing titer in BAL was low for both pH1N1 and H3N2 with no difference between groups (**Figures 2F, G** **)**.

In summary, after pH1N1 pre-exposure, IM immunization with ChAdOx2-NPM1-NA induced high serum IgG titers against both pH1N1 and H3N2, while AE delivery induced high IgG and IgA titers in BAL and nasal swabs. A significant increase in the serum-neutralizing H3N2 Ab titer was detected only in the IM group.

### Cytokine Responses in PBMC and Tissues

IFNγ ELISpot was performed to assess the cytokine-producing cells in PBMC, spleen, BAL, and tracheobronchial, prescapular, and retropharyngeal lymph nodes, draining the sites of immunization. Responses were evaluated following stimulation with pH1N1 and H3N2 live viruses or peptides covering the NP, M1, and NA proteins present in the vaccine. After pH1N1 challenge, the first IFNγ responses to pH1N1, H3N2, M1, and NP in PBMC were detected at 7 DPI as previously reported (36, 42).

**Figure 3:**
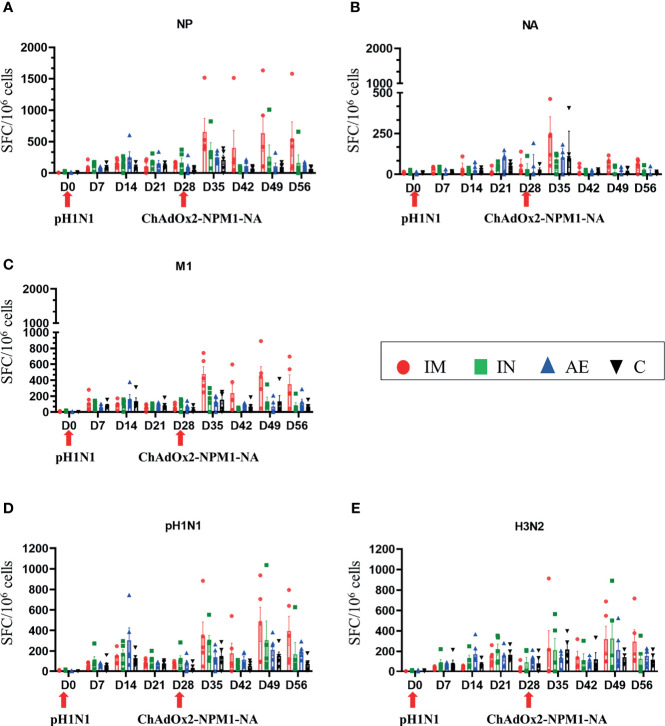
IFNγ ELISpot responses in PBMC. IFNγ secreting spot-forming cells (SFC) were enumerated during the time course following stimulation with a pool of peptides covering **(A)** NP, **(B)** M1, and **(C)** NA proteins or **(D)** pH1N1 and **(E)** H3N2 viruses. The arrows below D0 and D28 indicate the challenge of the pigs with pH1N1 and the immunization with ChAdOx2-NPM1-NA, respectively. The C group was not immunized. Each symbol represents an individual animal, the top of the bar represents the mean, and the line the standard error (SEM) except for graph **(B)**, where the top of the bar indicates the median and the line the interquartile range. Significant statistical differences are listed in **Table 2, 3.**.

IM immunization significantly increased the response in PBMC to NP (mean 653 SFC/10^6^ cells at 35 DPI), M1 (477 SFC/10^6^ cells at 35 DPI), pH1N1 (460 SFC/10^6^ cells at 49 DPI), and H3N2 (321 SFC/10^6^ cells at 49 DPI) (**Figures 3A, C–E**, respectively). IM immunization also induced the greatest response to NA 7 days post-immunization (mean 222 SFC/10^6^ cells at 35 DPI), although this rapidly declined and no significant differences were found (**Figure 3B**), in contrast to the NP, M1, and pH1N1 responses which were maintained until 56 DPI. Statistically significant differences were not observed for H3N2-stimulated cells as well. The responses to NP, M, and NA in control animals reached a peak between day 21 and day 35 as previously shown with experimental influenza infection in pigs (36, 42). Significant responses were reached at different time points after immunization and indicated in **Tables 2**, **3**. All groups made a cross-reactive response to H3N2, but there were no significant differences between groups (**Figure 3E**).

**Figure 4:**
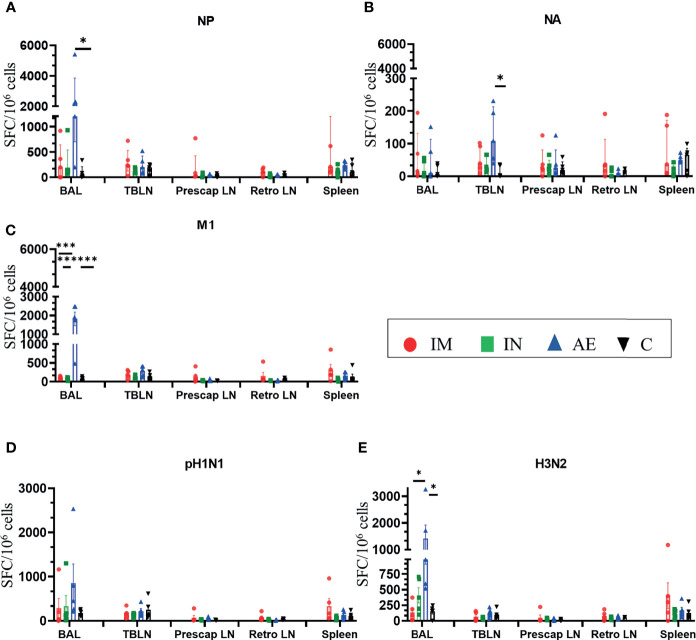
IFNγ ELISpot responses in tissues. IFNγ secreting spot-forming cells (SFC) were enumerated in BAL, tracheobronchial lymph nodes (TBLN), prescapular lymph nodes (prescap LN), retropharyngeal lymph nodes (retro LN), and spleen on D56. Cells from tissues were stimulated with a pool of peptides covering **(A)** NP, **(B)** M1, and **(C)** NA proteins or **(D)** pH1N1 and **(E)** H3N2 viruses. Each symbol represents an individual animal. For graphs **(A)** and **(B)**, Kruskal–Wallis and Dunn’s multiple comparisons tests were performed, and the top of these bars indicates the median and the line the interquartile range. The rest of the graphs were analyzed by one-way ANOVA and Bonferroni’s multiple comparisons test, and the top of the bar indicates the mean and the line the standard error (SEM). Asterisks denote significance between indicated groups (*p < 0.05, ***p < 0.001).

AE immunization induced significantly greater BAL responses to M1 compared to the other groups (**Figure 4C**), to NP compared to the control group (**Figure 4A**), and to H3N2 compared to the IM and control groups (**Figure 4E**). The response to NA was the highest in the AE group in tracheobronchial lymph nodes (**Figure 4B**). IM immunization induced a high response in the spleen, but the increase was not significantly different compared to the other groups and was only measured at one time point, in contrast to PBMC responses (**Figures 4A–E**). IN immunization did not induce a significant immune response compared to the other groups.

**Figure 5:**
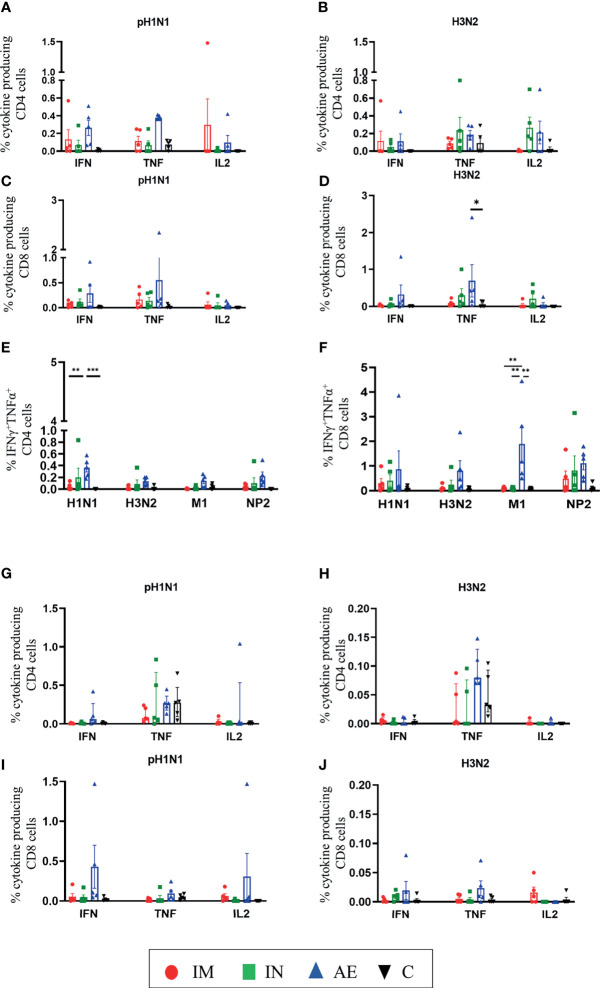
T-cell cytokine responses in BAL and TBLN. **(A–F)** BAL and **(G–J)** TBLN were collected 4 weeks post-immunization. Cryopreserved cells were thawed and stimulated with **(A, C, G, I)** pH1N1 or **(B, D, H, J)** H3N2, and IFNγ, IL-2, TNF, and IFNγTNF cytokine secretions were measured in CD4 and CD8 T cells by intracellular cytokine staining. IFNγTNF co-production within **(E)** CD4 and **(F)** CD8 T cells in BAL was determined following pH1N1, H3N2, or M1 and NP2 protein stimulation. Each symbol represents an individual animal. For graphs **(G)** and **(H)**, Kruskal–Wallis and Dunn’s multiple comparisons tests were performed, and the top of these bars indicates the median and the line the interquartile range. The rest of the graphs were analyzed by two-way ANOVA and Bonferroni’s multiple comparisons test, and the top of these bars indicates the mean and the line the standard error (SEM). Asterisks indicate significant differences (*p < 0.05, **p < 0.01, ***p < 0.001).

**Figure 6:**
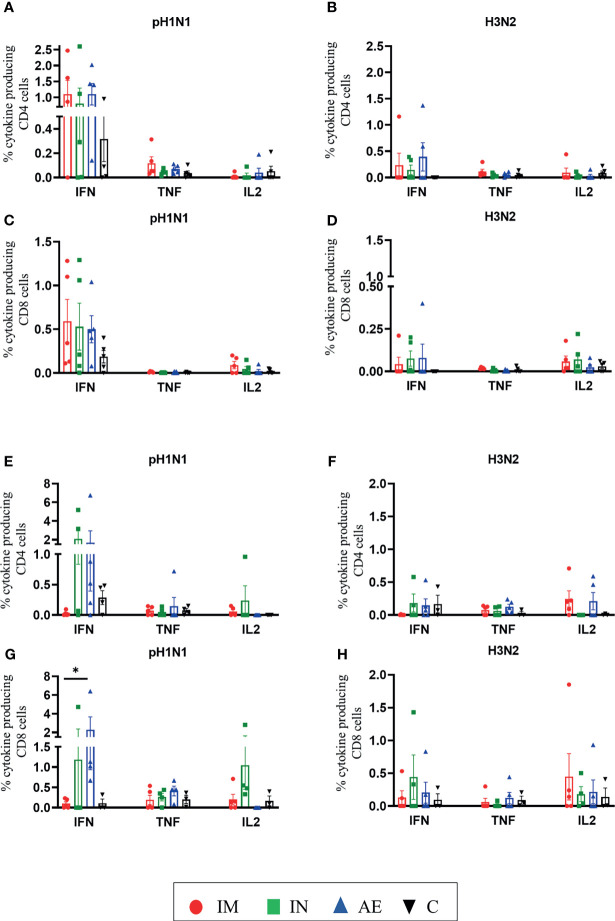
T-cell cytokine responses in the spleen and nasal turbinates. **(A–D)** Spleen and **(E–H)** nasal turbinates were collected at 4 weeks post-immunization. Cryopreserved cells were thawed and stimulated with **(A, C, E, G)** pH1N1 or **(B, D, F, H)** H3N2, and IFNγ, TNF, and IL-2 cytokine secretions were measured in CD4 and CD8 T cells by intracellular cytokine staining. Each symbol represents an individual animal; the top of the bar indicates the mean and the line the standard error mean (SEM). Two-Way ANOVA and Bonferroni multiple comparisons tests were used to compare responses between groups, and asterisks indicate significant differences (*p < 0.05).

We also analyzed IFNγ, TNF, and IL-2 production of CD8β and CD4 T cells by intracellular cytokine staining following *in vitro* stimulation with pH1N1, H3N2, NP, and M1. The single cytokine producers were defined as indicated in **Supplementary Figure 2**—gate 2 for IFNγ, gate 1 for TNF, and gate 4 for IL-2. Triple cytokine-producing cells (IFNγ/TNF/IL2) were not identified in any tissue analyzed, and only BAL contained significant frequencies of double producers which are shown in **Figures 5E, F**. AE immunization was the only regime that induced statistically higher responses compared to either control or IM groups in different tissues: H3N2-specific CD8 TNF in BAL (**Figure 5D**), pH1N1-specific double TNF/IFNγ-producing CD4 cells in BAL (**Figure 5E**), M1-specific double TNF/IFNγ-producing CD8 cells in BAL (**Figure 5F**), and pH1N1-specific CD8 IFNγ producers in nasal turbinates (**Figure 6G**). It is also apparent that IFNγ production is dominant in the spleen and nasal turbinates (**Figure 6**), while the BAL and TBLN responses are more balanced (**Figure 5**). These data indicate that IM immunization induced a strong IFNγ response in PBMC, while AE induced the highest IFNγ response and IFNγ/TNF co-producing cells in the BAL. The greatest response was to NP, followed by M1 with the lowest response to NA. IN and AE delivery induced comparable IFNγ response in nasal turbinates.

## Discussion

To mimic the effect of pre-existing immunity on vaccine-induced immune responses, we exposed pigs to pH1N1, which maintains antigenic similarity to human seasonal strains and provides a unique opportunity to use a virus affecting both humans and swine. We showed that ChAdOx2-NPM1-NA induced T-cell and Ab responses after pH1N1 pre-exposure. We evaluated the importance of the route of immunization and targeting different regions of the respiratory tract on the magnitude and nature of immune responses. We used IN delivery with a mucosal atomization device to restrict the antigen to the URT and used AE delivery by a vibrating mesh nebulizer to distribute the vaccine throughout the LRT and URT (33). The respiratory tract was compared to IM administration, the most widely used route of vaccine delivery. We showed that IM immunization after pH1N1 pre-exposure boosted blood T-cell and Ab responses but had a weak effect on the BAL response. In contrast, AE immunization boosted local BAL T-cell and Ab responses, but had no effect on the blood response, as we have previously observed with a different vaccine candidate (35). It should be noted that AE immunization delivers only a third of the dose so that this route appears to be extremely efficient in inducing immune responses (33). IN immunization increased the pH1N1-specific T-cell response in the nasal turbinates and spleen only marginally.

It was important to determine whether pre-exposure to conserved proteins such as NP and M1 (97% and 99% similarity between strains) interfered with the immune response to an antigen, NA (43% similarity between strains), to which the animals had not been previously exposed. This did not seem to be the case as the animals generated an anti-N2 antibody response, which could have contributed to the neutralization of H3N2. IFNγ and TNF T-cell responses against NP and M1 were significantly boosted in the blood and BAL, while there was an anti-NA response in TBLN 4 weeks post-AE immunization and transient response in PBMC 1 week after IM immunization, although this was weaker than the NP and M1 response. The possibility of response against the glycine linker, which was present in the NP and M1 pools, was not formally tested here. Therefore, pH1N1 pre-exposure did not appear to hinder responses induced by ChAdOx2-NPM1-NA.

Few studies have evaluated the immunological impact of prior influenza exposure on vaccine efficacy in large animal models. Chepkwony et al. demonstrated that prior H3N2 exposure followed by intramuscular immunization with whole inactivated heterologous H3N2 vaccine induced stronger and broader antibody responses (43). Ferrets with pre-existing immune responses influenced recombinant H2 antibodies following vaccination (44). In humans, the first exposure to influenza virus biases the subsequent responses to heterologous strain and the breadth of cross-reactivity (1–4). This may partly explain the variable efficacy of traditional, intramuscular-inactivated seasonal human influenza vaccine which provides between 10% and 60% protective efficacy. Furthermore, prior vaccinations can have a significant negative impact on antibody binding, antibody affinity maturation, and hemagglutinin inhibition responses to H1N1, H3N2, and B strains by inactivated vaccine platforms (5, 45). The response to N2 reported here may suggest that using a viral vector (ChAdOx2) may circumvent the poor response to heterologous antigen.

In humans, an alternative immunization strategy is the use of LAIV administered intranasally with an efficacy of 75%–80% in children, which induces a wider range of cellular, humoral, and mucosal immune responses than the inactivated vaccine (46). Pre-existing immunity, due to natural exposure or prior vaccination, may significantly impact the ability of the LAIV vaccine strain to replicate and therefore impair vaccine efficacy (47). This is supported by the observations that LAIV is less effective in young adults than children and ineffective in adults >50 years (46). A clinical trial in Bangladesh correlated higher pre-existing baseline antibodies derived from natural influenza A/H3N2 and B infections with low viral shedding/replication of LAIV (48). The strong responses we detect in IM and AE animals suggest that ChAdOx2-NPM1-NA is not easily inhibited by prior responses to the influenza.

It is not known whether it is important to target different regions of the respiratory tract to induce optimal protection against different respiratory infections. Restricting the response to the URT, by administering a smaller volume intranasally, as in the case of LAIV, may not be optimal for lung protection, as studies in mice and ferrets suggest that induction of cross-protective immunity against different types of influenza viruses is achieved most efficiently following vaccine delivery to the LRT (25, 49). However, a barrier to delivering existing LAIV to the LRT is that LAIV retains some potential to replicate, raising safety concerns for lung delivery (21). However, this is not a problem for replication-deficient viral vectored vaccines such as ChAdOx2-NPM1-NA. Therefore, it remains to be formally tested in challenge studies whether lung or URT targeting would be most effective for a vaccine directed against a respiratory pathogen. Additionally, combining systemic and respiratory immunization maybe a promising strategy requiring further investigation (35).

With regard to the repeated use of ChAdOx2, it has become clear from the COVID trials that adenoviral vectors can be used repeatedly in the same individuals. Anti-vector antibodies raised after the first immunization had minimal effect against the response to the second, and anti-vector antibodies do not increase with the second dose (50). Also, a third dose boosts responses further (51). Studies of the oral administration of Ad5 done by VaXArt also showed that no anti-vector immunity is induced and the same may be the case for other mucosal routes of immunization (52).

Respiratory viruses are among the greatest threat to global health. Therefore, there is an urgent need to better understand the mechanism of protective immunity to respiratory infections and to develop better animal models to test the efficacy of novel vaccines and therapies. Mice, guinea pigs, and ferrets are widely used for influenza virus research, but none of these small animal models accurately reflect the immune response in humans, particularly humans with pre-existing immunity. Pigs are an important natural host since they are susceptible to infection with many human seasonal strains and are a source of new human pandemic viruses. In this study, we demonstrated distinct immune responses to our candidate vaccine as a result of immunization route. We chose a pig model with pre-exposure to a heterologous influenza strain to best mimic adult human influenza A virus exposure. While we showed that the route of immunization had a significant impact on the type of immune response generated, it remains to be seen which may correlate best with protective efficacy. We demonstrate that ChAdOx2-NPM1-NA was immunogenic after pre-exposure, resulting in both T-cell immunogenicity and anti-NA antibody generation. We propose that the pig is a powerful model to dissect systemic and respiratory tract immune responses after influenza pre-exposure and immunization. Our data suggest that the immune responses arising from multiple routes of administration of ChAdOx2-NPM1-NA warrant further study to determine protective efficacy. These studies will provide valuable insight into the development of universal influenza and other respiratory virus vaccines and inform future vaccine and clinical trial design.

## Data Availability Statement

The original contributions presented in the study are included in the article/**Supplementary Material**. Further inquiries can be directed to the corresponding authors.

## Ethics Statement

The swine animal experiment was approved by the ethical review process at APHA and followed the UK Government Animal (Scientific Procedures) Act 1986. Mice were used in accordance with the UK Animals (Scientific Procedures) Act 1986 under project license number P9804B4F1 granted by the UK Home Office with approval from the local Animal Welfare and Ethical Review Board (AWERB) at the University of Oxford.

## Author Contributions

ET, SG, and TL designed the study and obtained the funding. EA, SM, RK, MU, and AB generated the vaccine and carried out the mouse experiments. EV, TM, AM, VC, and RM designed and performed the pig experiments. EV, TM, ET, AM, VM, BP, VC, TC, and EM processed the samples. EV, TM, and AM analyzed the pig data. PD and HE oversaw the pig studies and infected and immunized the animals. ET, EV, and EA wrote the manuscript. All authors contributed to the article and approved the submitted version.

## Funding

This work was supported by the Medical Research Council (grant number MR/S037160/1) and UKRI Biotechnology and Biological Sciences Research Council (BBSRC). It was also supported by the Institute Strategic Programme and Core Capability Grants to The Pirbright Institute (BBS/E/I/00007031, BBS/E/I/00007037, BB/S506680/1, and BBS/E/I/00007039).

## Conflict of Interest

SG is co-founder of Vaccitech and named as an inventor on a patent covering use of ChAdOx2-vectored vaccines. TL is a consultant to Vaccitech. RM is an employee of Aerogen Limited and is a named inventor on inhaled vaccine delivery system patents.

The remaining authors declare that the research was conducted in the absence of any commercial or financial relationships that could be construed as a potential conflict of interest.

## Publisher’s Note

All claims expressed in this article are solely those of the authors and do not necessarily represent those of their affiliated organizations, or those of the publisher, the editors and the reviewers. Any product that may be evaluated in this article, or claim that may be made by its manufacturer, is not guaranteed or endorsed by the publisher.
