# Supplementary material for: Respiratory and Intramuscular Immunization With ChAdOx2-NPM1-NA Induces Distinct Immune Responses in H1N1pdm09 Pre-Exposed Pigs
Source: Front Immunol. 2021 Nov 3;12:763912. doi: 10.3389/fimmu.2021.763912 (PMC8595216; doi:10.3389/fimmu.2021.763912)
Supplement: Supplementary file 1 [file DataSheet_1.pdf]

Supplementary Material

1 Supplementary Data

Mice Immunogenicity Supplementary Methods

1.1 Murine immunogenicity model

Ethics statement

Mice were used in accordance with the UK Animals (Scientific Procedures) Act 1986 under project license number P9804B4F1 granted by the UK Home Office with approval from the local Animal Welfare and Ethical Review Board (AWERB) at the University of Oxford. Age matched animals were purchased from commercial suppliers and randomly split into groups on arrival at our facility. Animals were group housed in IVCs under SPF conditions, with constant temperature (20-24°C) and humidity (45-65%) with lighting on a 13:11 light-dark cycle (7am to 8pm). For induction of short-term anaesthesia, animals were anaesthetized using vaporised IsoFlo®. All animals were humanely euthanized at the end of each experiment by an approved Schedule 1 method.

Immunizations

Inbred BALB/cOlaHsd (BALB/c) (Envigo) (n=5 mice per group) and outbred CD1 (Envigo), were immunized intramuscularly (IM) in the musculus tibialis with 8 x 10^7 infectious units (IU) of ChAdOx2 vector (either ChAdOx2-NPM1, ChAdOx2-NA+NPM1, or ChAdOx2-NA). Three weeks after the immunization mice were euthanized. Serum and spleen samples were collected for assessment of humoral and cell mediated immunity.

1.2 Mouse- Total IgG neuraminidase ELISA

Recombinant NA (sequence matched to the vaccine antigen, Genbank accession number: ATE49827) was produced by The Native Antigen company. MaxiSorp plates (Nunc) were coated with 100 ng/well NA overnight at 4 ^{o}C prior to washing in PBS/Tween (0.05% v/v) and blocking with Blocker Casein in PBS (Thermo Fisher Scientific) for 1 h at room temperature (RT). A reference standard positive serum pool was generated with mouse serum with high endpoint titre. The reference standard, individual mouse serum samples, negative and an internal control (diluted in casein) were incubated for 1 h at RT. Following washing, bound antibodies were detected by addition of a 1 in 5000 dilution of alkaline phosphatase (AP)-conjugated goat anti-mouse IgG (Sigma-Aldrich) for 1 h at RT and addition of p-Nitrophenyl Phosphate, Disodium Salt substrate (Sigma-Aldrich). An arbitrary number of ELISA units (EU) were assigned to the reference pool and optical density values of each dilution were fitted to a 4-parameter logistic curve using GraphPad Prism software. All data were log-transformed for presentation and statistical analyses.

1.3 NPM1 IFNγ ELISpot

Spleen cells were passed through 70µM cell strainers to obtain a single cell suspension. The cell suspension was then treated with ammonium potassium chloride lysis solution and resuspended in alpha MEM supplemented with 10% FCS, penicillin/streptomycin and L-glutamine. Splenocytes were added to hydrophobic-PVDF ELISpot plates (Merck) coated with 5µg/mL anti-mouse IFNγ and stimulated with NP1, NP2 and M1 peptide pools at a final concentration of 2µg/mL **(Supplementary Table 1)** for 18 hours at 37°C. Spot forming cells were detected by staining with anti-mouse IFNγ biotin (1mg/mL) followed by streptavidin-Alkaline Phosphatase (1mg/mL) with final development with AP conjugate substrate kit (BioRad, UK). Spots were counted using an AID ELISpot reader and software. IFNg spot forming units (SFU) were calculated by subtracting spots counted in from unstimulated splenocytes from the peptide stimulated wells. Data is represented as pooled SFU from NP1, NP2 and M1 peptide pools.

2 Supplementary Tables and Figures

Supplementary Table 1. Pools of peptides (15mers overlapping by 10) for each antigen were prepared as detailed.

Pool	Genbank Accession number	Amino acid residues	Number of peptides
NA1	ATE49827	1-239	57
NA2	ATE49827	227-469	58
NP1	AKJ82485	1-259	62
NP2*	AKJ82485	248-498	62
M1*	KR701100.1	1-252	61

*These pools include the glycine linker region between NP and M1

.

2.1 Supplementary Figures

Supplementary Figure 1

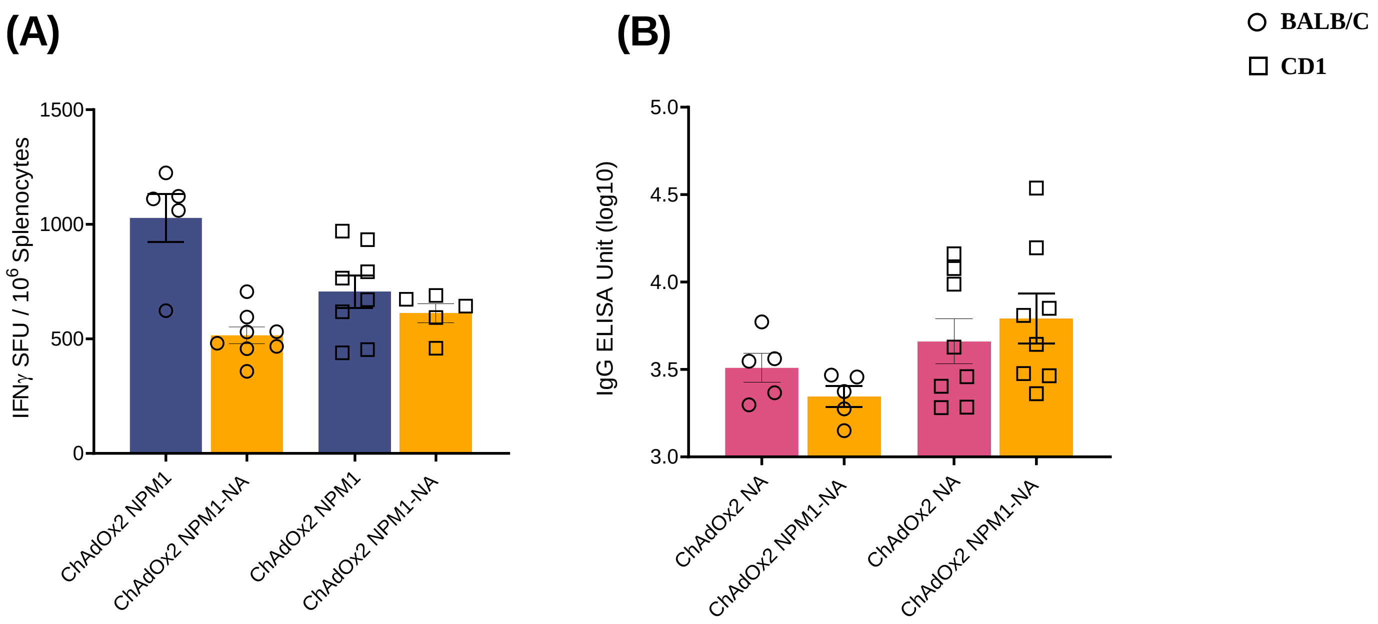

Supplementary Figure 1. Bivalent ChAdOx2 vectored vaccine produces robust humoral and cellular immune responses in mice, comparable with monovalent ChAdOx2 vaccines. Three groups of either inbred Balb/c mice (circles) or outbred CD1 mice (squares) were vaccinated (IM) with 8 x10^7 IU units of either ChAdOx2-NA, ChAdOx2-NPM1 or the bivalent vaccine ChAdOx2-NA+NPM1 and 21-day post-vaccination (dpv) immune responses were assessed. **(A)** Summed IFNγ ELISpot responses in splenocytes to peptides that spanned the NPM1 fusion protein (pooled NP1, NP2 and M1 **Supplementary Table 1**) at 21 dpv with ChAdOx2 NPM1 or ChAdOx2 NA+NPM1. ChAdOx2-NA vaccinated mice did not show a positive response (data not shown). **(B)** Standardized total IgG ELISA was used to detect humoral responses to NA at 21 days after vaccination with ChAdOx2 NA or ChAdOx2 NA+NPM1. No positive responses were detected in the ChAdOx2-NPM1 groups (data not shown). Data were analyzed by Mann-Whitney test and no significant differences ($P<0.05$) were detected between the bivalent and monovalent vaccines.

Supplementary Figure 2

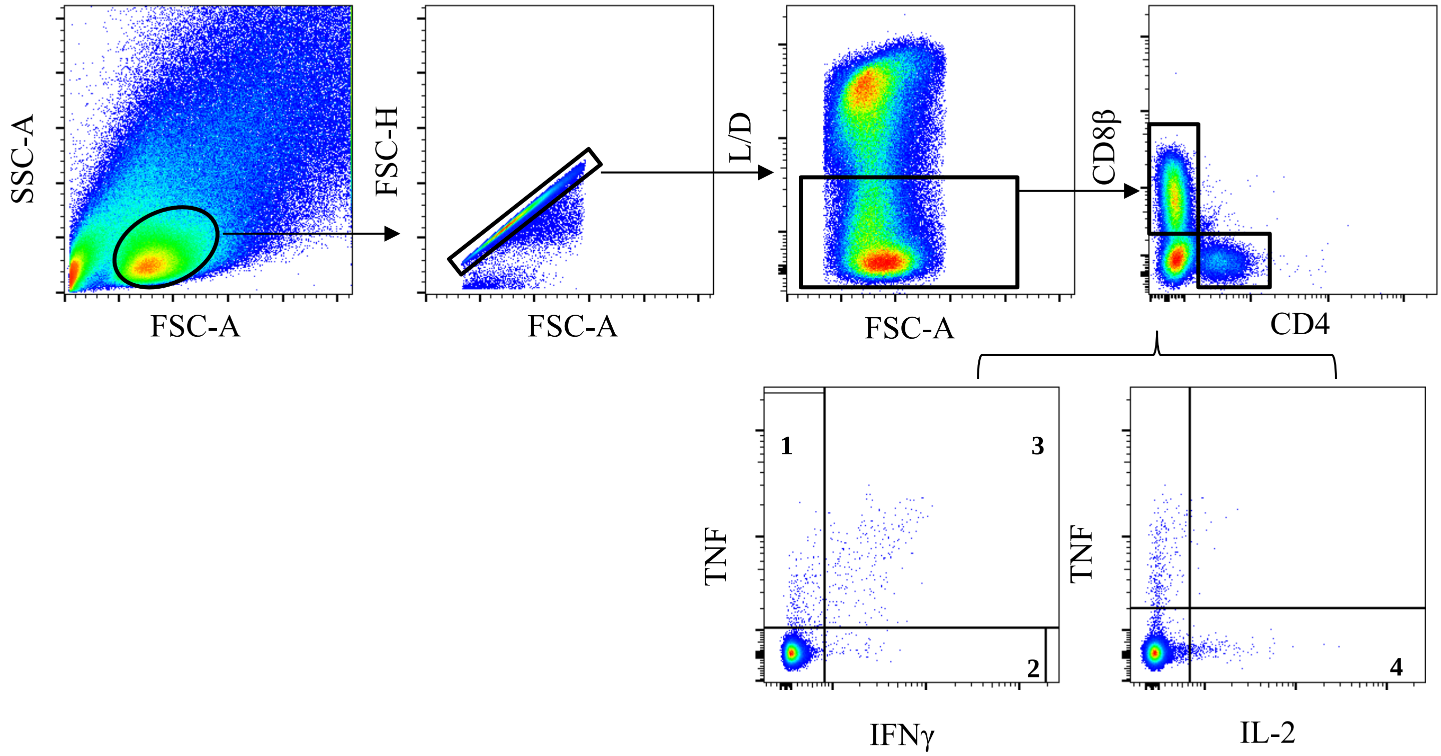

Supplementary Figure 2. Gating strategy followed for analysis of cytokine production by porcine CD4 and CD8 T cells. Cells were stimulated overnight with pH1N1 or H3N2 or for 5 hours with NP2 or M1, followed by intracellular cytokine staining. Lymphocytes were gated by light scatter and were further sub-gated for exclusion of doublets and dead cells. Live cells were gated for CD4 and CDβ8 cells and production of TNF (1), IFNγ (2), TNF/IFNγ (3) and IL-2 (4) was determined with the indicated gates. The gates shown are from one representative animal from BAL tissue stimulated with pH1N1.

.
